# The Interplay between Mucosal Microbiota Composition and Host Gene-Expression is Linked with Infliximab Response in Inflammatory Bowel Diseases

**DOI:** 10.3390/microorganisms8030438

**Published:** 2020-03-20

**Authors:** Nikolas Dovrolis, George Michalopoulos, George E. Theodoropoulos, Kostantinos Arvanitidis, George Kolios, Leonardo A. Sechi, Aristidis G. Eliopoulos, Maria Gazouli

**Affiliations:** 1 Laboratory of Pharmacology, Faculty of Medicine, Democritus University of Thrace, Alexandroupolis 68100, Greece; ndovroli@med.duth.gr (N.D.); karvanit@med.duth.gr (K.A.); gkolios@med.duth.gr (G.K.); 2 Gastroenterology Department, Tzaneion General Hospital, Piraeus 18536, Greece; gmicha78@hotmail.com; 3 1st Propaedeutic University Surgery Clinic, Hippocratio General Hospital, Medical School, National and Kapodistrian University of Athens, Athens 11527, Greece; georgetheocrs@live.com; 4 Department of Biomedical Sciences, University of Sassari, Sassari 07100, Italy; sechila@uniss.it; 5 Laboratory of Biology, Medical School, National and Kapodistrian University of Athens, Athens 11527, Greece; eliopag@med.uoa.gr; 6 Centre of Basic Research, Biomedical Research Foundation of the Academy of Athens (BRFAA) 11527, Greece

**Keywords:** inflammatory bowel disease, infliximab, microbiota, microbiome, anti-TNF, response to therapy, host transcriptome, biomarkers

## Abstract

Even though anti-TNF therapy significantly improves the rates of remission in inflammatory bowel disease (IBD) patients, there is a noticeable subgroup of patients who do not respond to treatment. Dysbiosis emerges as a key factor in IBD pathogenesis. The aim of the present study is to profile changes in the gut microbiome and transcriptome before and after administration of the anti-TNF agent Infliximab (IFX) and investigate their potential to predict patient response to IFX at baseline. Mucosal biopsy samples from 20 IBD patients and nine healthy controls (HC) were examined for differences in microbiota composition (16S rRNA gene sequencing) and mucosal gene expression (RT-qPCR) at baseline and upon completion of IFX treatment, accordingly, via an in silico pipeline. Significant differences in microbiota composition were found between the IBD and HC groups. Several bacterial genera, which were found only in IBD patients and not HC, had their populations dramatically reduced after anti-TNF treatment regardless of response. Alpha and beta diversity metrics showed significant differences between our study groups. Correlation analysis revealed six microbial genera associated with differential expression of inflammation-associated genes in IFX treatment responders at baseline. This study shows that IFX treatment has a notable impact on both the gut microbial composition and the inflamed tissue transcriptome in IBD patients. Importantly, our results identify enterotypes that correlate with transcriptome changes and help differentiate IFX responders versus non-responders at baseline, suggesting that, in combination, these signatures can be an effective tool to predict anti-TNF response.

## 1. Introduction

Inflammatory bowel diseases (IBD) encompass Crohn’s disease (CD) and ulcerative colitis (UC), which are chronic, immune-mediated pathologies. Whilst their exact etiologies remain elusive, both genetic and environmental factors have been implicated in the establishment of systemic inflammatory reactions that typify IBD [1,2]. A major, modifiable environmental factor is the gut microbiota [3]. Imbalances in microbiota composition have been linked to IBD pathogenesis in both humans [4] and mouse models of IBD [5] and associated with both the onset and progression of the disease [6,7,8]. The intestinal microbiota affects the function of several immune cell types, including regulatory T cells (Tregs) that orchestrate local immune reactions and maintain gut homeostasis [9]. Disruption of host and microbial homeostasis instigates deregulated immune reactions which elicit pro-inflammatory programs driving IBD. Among them, the pro-inflammatory cytokine tumour necrosis factor (TNF) has a major role and is a target for IBD therapy [10].

Indeed, anti-TNF agents exhibit efficacy against both CD and UC, sustaining clinical and endoscopic remission and improving overall clinical benefits [11]. However, these therapies are associated with unsatisfactory remission rates that result from primary non-response (20%–40% in clinical trials) [12] or from loss of response, frequently, due to immunogenicity and increased anti-TNF clearance, in approximately 13%–24% of patients after one year [13]. Additionally, the treatment is associated with harmful side effects [14] and is considered expensive [11]. Several studies have previously explored gene expression signatures for the prediction of anti-TNF response and proposed different sets of putative biomarkers [15,16,17].

Anti-TNF agents may modify intestinal microbiota by both direct and/or indirect pathways. Recent data by Aden et al. [18] supported that anti-TNF therapy shifted the diversity of fecal microbiota in patients with IBD, but diversity indices did not vary significantly between responders and non-responders. Anti-TNF based treatments heal and down-regulate inflammation in the damaged intestinal mucosa, thereby restoring normal structure of the intestinal epithelium [19] and controlling tolerance functions toward mucosal microbiota. Thus, anti-TNF agents could indirectly change microbiota composition. It has been suggested that by modulating the gut microbiota and by inducing T cell apoptosis and inhibiting vasculitis, TNF inhibition can limit inflammation in IBD patients [20,21]. Recently, it was reported that reduced Firmicutes abundance is linked with a shorter time to relapse after infliximab (IFX) withdrawal [22]. Furthermore, Kolho et al. [23] showed that the increased abundance of six clades of bacteria, including *Eubacterium rectale* and *Bifidobacterium* spp., can predict the response to anti-TNF therapies in pediatric IBD patients. These studies indicated that the gut microbiota may provide possible biomarkers for monitoring and predicting IBD treatment outcomes.

The content and distribution of bacterial communities differ along the GI tract [24]. However, it is currently unknown whether IBD and the available therapeutic regimens would modify the composition of the gut microbiota in a constant way independently of topological influences. To this end, we herein focus on mucosal biopsy samples to investigate changes in the intestinal microbiota that could be most relevant to the response to IFX at baseline and after 3 months of treatment. Furthermore, via a combined microbiome–host gene expression correlation analysis, we aimed to establish the combined power of microbiota composition and transcriptional changes in predicting clinical response to treatment.

## 2. Materials and Methods

### 2.1.Samples

In total, 43 mucosal biopsy samples were obtained from the rectum during colonoscopy from 29 individuals [14 CD patients, 6 UC patients and 9 healthy controls (HC)]. All biopsies were immediately placed in Allprotect Tissue Reagent (Qiagen, Hilden, Germany) and stored according to manufacturer’s instructions. Of these samples, 28 are pairs before and after anti-TNF treatment (10 CD patients and 4 UC) and were used to study the treatment’s effects on the microbiome, as well as to find putative microbial biomarkers predicting treatment response (for CD we had 5 responders and 5 non-responders and for UC 2 responders and 2 non-responders). Finally, 4 CD and 2 UC patients were never issued an anti-TNF treatment and their samples were used only for microbiome differential analysis between IBD and HC to provide us with a larger pool sample for studying dysbiosis during IBD.

IBD diagnosis was based on standard clinical, endoscopic, radiological, and pathological criteria [25]. IFX was administered intravenously at a dose of 5 mg/kg at weeks 0, 2, 6 and every 8 wks thereafter. Patients that received other IBD treatments, were younger than 18 years in age, had used antibiotics or probiotics within the previous 6 weeks, had other known chronic disease, and were on pregnancy or breastfeeding status were excluded from the study.

The clinical and endoscopic disease activities were determined using the Mayo scoring system [26], the Harvey–Bradshaw Index (HBI) and C-reactive protein (CRP), respectively, at various time points—at baseline (before 1st infusion or injection), the day before each subsequent drug administration and at week 12 of treatment—were also assessed where appropriate (Supplementary Table S1). Ileocolonoscopy was performed, at baseline and after 12-20 wk of therapy, to assess mucosal healing. Changes to clinical and endoscopic imaging, compared to baseline, were classified in four categories and patients were classified as responders or not to anti-TNF therapy as previously described [27]. The Ethics Committee of Medical School of National and Kapodistrian University approved this study and the patients were included in the study after providing written consent.

### 2.2. RNA Extraction and Gene Expression

RNA extraction was performed from mucosal biopsies during diagnostic colonoscopy using the Qiagen AllPrep RNA/DNA Mini Kit (Qiagen, Hilden, Germany). cDNA was prepared using the RT2 First Strand Kit (Qiagen) according to the manufacturer’s instructions. Gene expression quantification was performed by RT2 profiler PCR Array Human Inflammatory Response and Autoimmunity (PAHS-077Z, Qiagen) on the same biopsy samples used for the microbiome analysis using the RT2 qPCR SYBR Green ROX Master Mix (Qiagen). Data were analysed in the RT2 Profiler PCR Array Data Analysis version 3.5 (Qiagen). All samples passed the quality checks for PCR Array reproducibility, RT efficiency, and genomic DNA contamination.

### 2.3. Differential Gene Expression Analysis

Using the RT2 Profiler PCR Array Gene Expression platform, differential gene expression analysis (DGEA) was performed amongst 8 sample groupings:

(1) Group 1: CD patients who responded to IFX treatment (5 samples at baseline)
(2) Group 2: CD patients who responded to IFX treatment (5 samples after treatment)
(3) Group 3: CD patients who did not respond to IFX treatment (5 samples at baseline)
(4) Group 4: CD patients who did not respond to IFX treatment (5 samples after treatment)
(5) Group 5: UC patients who responded to IFX treatment (2 samples at baseline)
(6) Group 6: UC patients who responded to IFX treatment (2 samples after treatment)
(7) Group 7: UC patients who did not respond to IFX treatment (2 samples at baseline)
(8) Group 8: UC patients who did not respond to IFX treatment (2 samples after treatment)

Pairwise DGEA was performed on the groups and Fold regulation was calculated using the 2^−ΔΔCt^ formula. ΔCt was calculated versus the Ct (Cycle threshold) values of the ACTB, B2M, GAPDH, HPRT1, RPLP0, HGDC housekeeping genes.

### 2.4. DNA Extraction and 16S rRNA Amplicon Sequencing

Total DNA was purified from the biopsies using the AllPrep DNA/RNA Mini Kit (Qiagen, Hilden, Germany) following the manufacturer’s instructions.

Sequencing services were performed by external independent facilities (MR DNA -Molecular Research LP, Shallowater, TX, USA). For sequencing, an Ion Torrent PGM was used with the 515/806 primers (Linker Primer Sequence: GTGYCAGCMGCCGCGGTAA, Reverse Primer Sequence: GGACTACNVGGGTWTCTAAT) on the V4 hypervariable region. The final fasta and quality files were converted into fastq files using software provided at http://www.mrdnafreesoftware.com/ (mrdna_fastqfastaqualconverter, mrdna_fastqprocessor) along with the conversion to QIIME2 [28] objects for further analysis.

### 2.5. Pre-Processing and OTU Picking

Qiime2 analysis was performed in-house using q2cli version 2019.1.0. The preprocessing steps included demultiplexing with the default parameters of demux, quality control and denoising using dada2 [29], and finally feature table and phylogenetic tree construction. The final feature table contained 2905 features with a total frequency of 2,976,482. The median frequency per sample was 61,552. The minimum sampling depth for all samples was 12,786. Finally, taxonomic classification was performed using a classifier trained on the SILVA [30] r132 99% OTU dataset, using scikit-learn 0.20.2 [31], specifically for the 515/806 primers.

### 2.6. Downstream Analysis

Downstream analysis was performed exclusively via the Calypso web platform v.8.84 [32]. During quality filtering, all taxa with less than 0.01 percent relative abundance across all samples were removed, the top 3000 taxa based on mean abundance were included and cyanobacteria and chloroplasts were excluded. Raw feature counts were transformed into relative abundance using total sum normalization (TSS) and SquareRoot (Hellinger) transformation. To identify microbiome differences between IBD patients and healthy individuals but also identify taxa possibly responsible for anti-TNF treatment response, we calculated and will be presenting the overall relative abundance for various sample groupings (before and after treatment); α-diversity (rarefied to a read depth of 12180) is the metric commonly used to identify dysbiosis during health conditions. In our case, using species evenness as criteria, we quantified the different numbers of microbial taxa in the samples study. Beta (β)-diversity, on the other hand, calculated here using the sPLS-DA method of mixMC [33] on the top 1000 OTUs, before and after treatment where applicable, is the metric which shows how qualitatively different the enterotypes are (the identities of taxa in each sample). We also explored differential relative abundance between sample groupings (using ANOVA), biomarker prediction via linear discriminant analysis effect size (LEfSe) [34], and core enterotype differences all on a genus level.

Finally, in order to identify correlations between the microbiome and specific inflammatory-response and autoimmunity related genes, revealing possible paired biomarkers regarding prediction of response to IFX treatment, we reduced the microbial data to include only the microbial genera identified by the differential abundance analysis between CD patients who will respond or not at baseline (Groups 1 and 3) and also only utilised the expression data of the statistically important genes (over absolute 2 fold change and *p* < 0.05). On these samples, Spearman’s rho correlation was performed between the microbial genera and the expression of the genes (from the Ct values) and visualised through heatmaps. As high Ct values indicate low expression of genes, we calculated Spearman’s rho between the microbial genera abundance and 1/Ct for each gene. Strong positive correlations highlight similar changes between microbial abundance and host gene expression (e.g., parallel induction or reduction), whereas negative correlations denote an inverse relationship (e.g., one is reduced while the other is increased).

**Figure 1:**
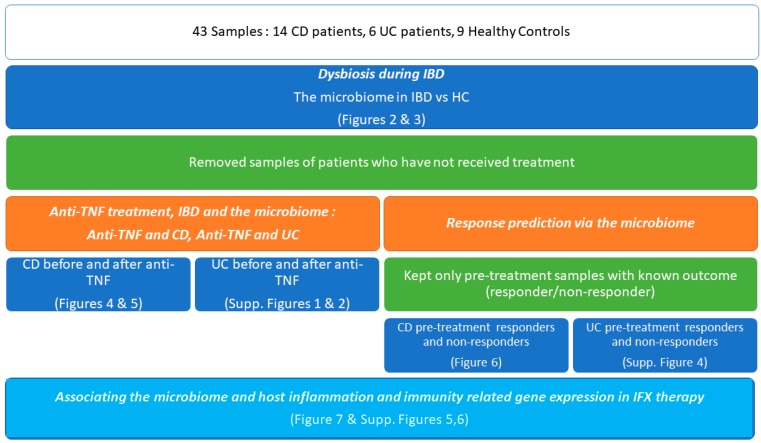
Overall design of the study. This protocol allowed us to showcase IBD vs healthy controls dysbiosis, to highlight the influence of Infliximab on microbiota composition and identify response-related microbial and transcriptional biomarkers.

Our complete approach is depicted in the flowchart of Figure 1.

## 3. Results

In order to appreciate the microbiota changes in IBD and its interactions with the anti-TNF treatment, we analysed mucosal biopsy samples and examined the data through different perspectives.

### 3.1. Dysbiosis during IBD

**Figure 2:**
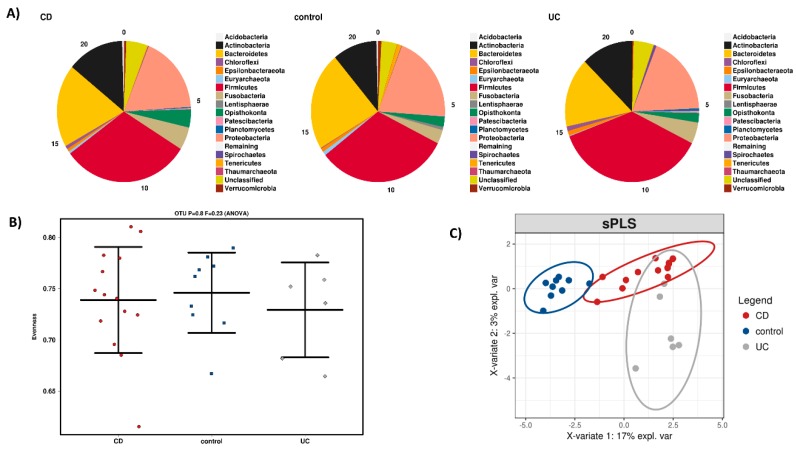
(**A**) Microbiota composition changes at phylum level among healthy controls, Crohn’s disease and ulcerative colitis patients. (**B**) α-diversity (quantification of biodiversity) differences in the 3 groups. (**C**) β-diversity (qualitative enterotype differences) of the 3 groups.

To explore the intestinal microbial landscape in Greek IBD patients, an overall abundance of microbial phyla was calculated as depicted in Figure 2A using biopsies from 14 CD and six UC patients versus nine healthy subjects served as controls (HC) who did not receive anti-TNF treatment. Although no statistically significant differences at phylum level were noted, a relative reduction in *Bacteroidetes* and an increase in *Actinobacteria*, *Fusobacteria* and *Chloroflexi* were detected in IBD versus HC biopsies. These increasing absences of statistically significant differences in microbial phyla populating mucosal intestinal tissue of CD patients have also been reported by Chiodini et al. [35] and may reflect geographical influences, as previously noted [36]. Figure 2B depicts the differences in α-diversity between the HC and the IBD phenotypes at the OTU level. Again, no statistically significant quantitative biodiversity differences were detected between sample groups. However, the microbiomes of UC, CD and HC display qualitative differences at the OTU level (Figure 2C).

**Figure 3:**
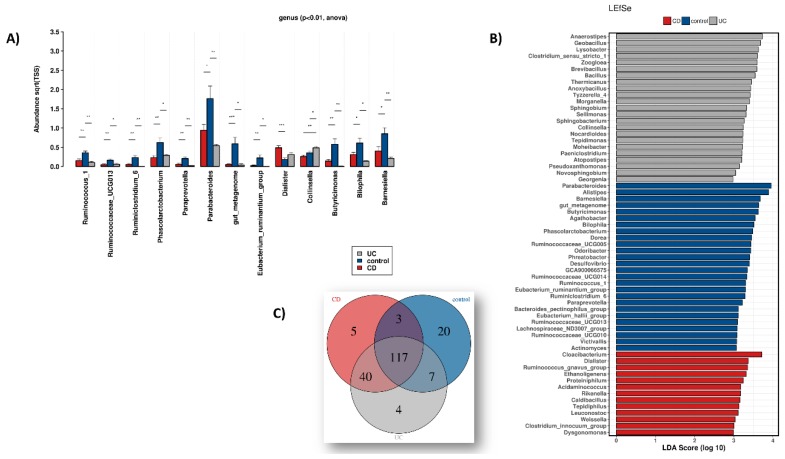
**(A**) Relative abundance changes of the microbial genera among healthy controls (control), Crohn’s disease (CD) and ulcerative colitis (UC) patients. (**B**) LEfSe analysis showing microbial genera associated with the 3 groups. (**C**) Venn diagram depicting the microbial genera constantly present (core microbiome) in the samples of the 3 groups versus those found exclusively in HC, CD and UC.

At genus level, statistically significant (*p* < 0.01) differential abundance between the HC and IBD groups was observed (Figure 3A). The genera *Parabacteroides*, *Barnesiella*, *Butyricimonas*, *Ruminococcus_1*, *Ruminococcaceae_UCG013*, *Phascolarctobacterium*, *Ruminoclostridium_6*, *Paraprevotella* as well as members of the *Eubacterium_ruminantium group* appear reduced in IBD, whereas *Dialister* abundance is increased. The genus *Collinsella* exhibits an induction during UC versus both HC and CD. Finally, we report the group of non-specific taxa (due to restrictions of the SILVA database) which also appear reduced in IBD.

A “biomarker-oriented” approach shown in Figure 3B that is based on linear discriminant analysis effect size (LEfSe) was performed and revealed several microbial genera associated with different groups. Overall, as shown in the Venn diagram of Figure 3C, there are 117 genera with high enough relative abundance shared between all three groups, 20 presents only in HC, five present only in CD and four present only in UC biopsies. A complete list of the genera depicted in this Venn diagram is provided in Supplementary Dataset S1.

### 3.2. Anti-TNF Treatment, IBD and the Microbiome

To study the effects of anti-TNF treatment on the microbiome, we analysed biopsies taken from CD and UC patients following treatment with IFX. We divided these samples into three categories for each IBD phenotype: responders to treatment (CD_R and UC_R), non-responders to treatment (CD_NONR and UC_NONR) and the biopsy samples pre-treatment (CD_PRE and UC_PRE).

**Figure 4:**
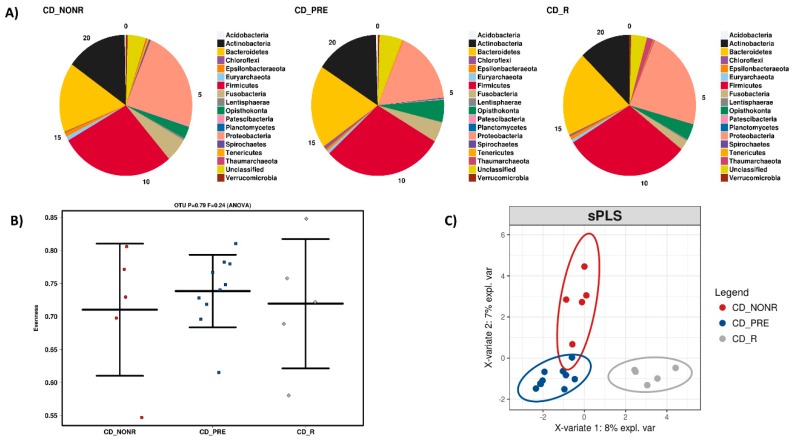
(**A**) Microbiota composition at phylum level among Crohn’s disease patients before treatment (CD_PRE) and after treatment in non-responders (CD_NONR) and responders (CD_R). (**B**) α-diversity (quantification of biodiversity) differences of the 3 groups. (**C**) β-diversity (qualitative enterotype differences) of the 3 groups.

No statistically significant changes were detected between groups for the overall abundance of microbial phyla in CD but a decrease in *Fusobacteria* and an increase in *Chloroflexi* were noted for responders, in parallel to an increase in *Proteobacteria* after treatment, regardless of response (Figure 4A). Alpha-diversity is reduced after anti-TNF treatment, without, however, reaching statistical significance (Figure 4B). Beta-diversity suggests differences before and after treatment as well as between responders and non-responders (Figure 4C).

**Figure 5:**
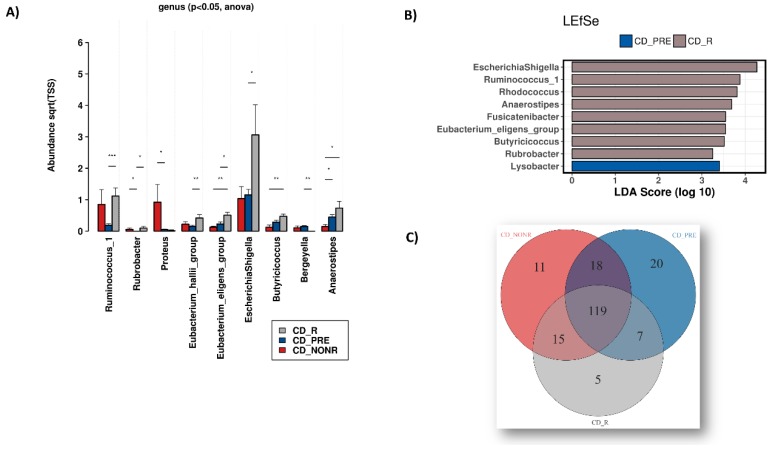
(**A**) Relative abundance of microbial genera among Crohn’s disease patients before treatment (CD_PRE) and after treatment in non-responders (CD_NONR) and responders (CD_R). (**B**) LEfSe analysis showcasing microbial genera associated with the sample groups. (**C**) Venn Diagram depicting the microbial genera constantly present (core microbiome) in the samples of the 3 groups versus those found only in responders, non-responders and at baseline (before initiation of treatment).

Differential abundance at genus level (Figure 5A) highlighted a statistically significant (*p* < 0.05) increase in *Rubrobacter* abundance after IFX treatment, regardless of response. Interestingly, *Proteus* and *Bergeyella* abundance increased in non-responders and responders to IFX, respectively. *Ruminococcus_1* increased in anti-TNF treated CD patients regardless of response but reached statistical significance only between responders and pre-treatment. The *Eubacterium_hallii*_group, *Eubacterium_eligens*_group, *Escherichia*, and *Shigella* populations also increased in responders versus pre-treatment samples. *Anaerostipes* are decreased in non-responders versus both the responders and the pre-treatment groups. Finally, *Butyricicoccus* became abundant in responders versus non-responders.

By using the biomarker discovery tool LEfSe, we identified microbial genera associated mainly with CD responders (Figure 5B). Further analysis identified 119 microbial genera that are common between groups, five found in responders, 11 in non-responders and 20 only in pre-treatment samples (Figure 5C). The complete list of genera shown in Figure 5C is provided in Supplementary Dataset S2.

In UC patients, overall phyla abundance (Supplementary Figure S1A) did not contain statistically significant changes but the effects of anti-TNF treatment are reflected by an induction of *Bacteroidetes* population in responders and a loss of some other phyla such as *Spirochaetes* and *Planctomycetes*. In the non-responders group, there is an increase in *Actinobacteria*. Alpha-diversity (Supplementary Figure S1B) showcases increased biodiversity in non-responders and a loss of OTUs in responders. Beta-diversity points to distinct enterotypes for the three groups before and after anti-TNF treatment (Supplementary Figure S1C).

By exploring the differential abundance of bacteria genera in these samples (Supplementary Figure S2A) we detected statistically significant (*p* < 0.01) differences in UC before and after treatment. Specifically, the populations of *Veillonela*, *Tyzerella*, *Ruminococcus_torques*_group, *Parabacteroides*, *Erisypelatoclostridium* and *Bilophila* are all significantly increased in responders to treatment versus either of the other groups, whereas *Porphyromonas*, *Granulicatella* and *Corynebacterium*_1 genera are increased in the non-responders group versus the other two. The LEfSe tests (Supplementary Figure S2B) linked *Bilophila* to responders and *Granulicatella* to non-responders. Analysis of the core microbiome of the sample groups (Supplementary Figure S2C) revealed 101 microbial genera present regardless of treatment, seven unique for the responders, eight unique for non-responders and 24 unique to pre-treatment UC samples. The complete list of these genera can be found in Supplementary Dataset S3.

An interesting finding of this study is the fact that there are eight bacterial genera that can be found only in IBD patients and not HC which are reduced to non-detectable levels after anti-TNF treatment regardless of response. These are *Zoogloea*, *Tepidiphilus*, *Rikenella*, *Proteiniphilum*, *Pedobacter*, *Morganella*, *Lysobacter* and *Caldibacillus*. Supplementary Figure S3 depicts the respective barplots between all sample groupings.

### 3.3. IFX Response Prediction via the Microbiome

To determine if gut microbiota could predict patient response to anti-TNF, we compared differences in microbial abundance at genus level between responders and non-responders, before initiation of treatment.

**Figure 6:**
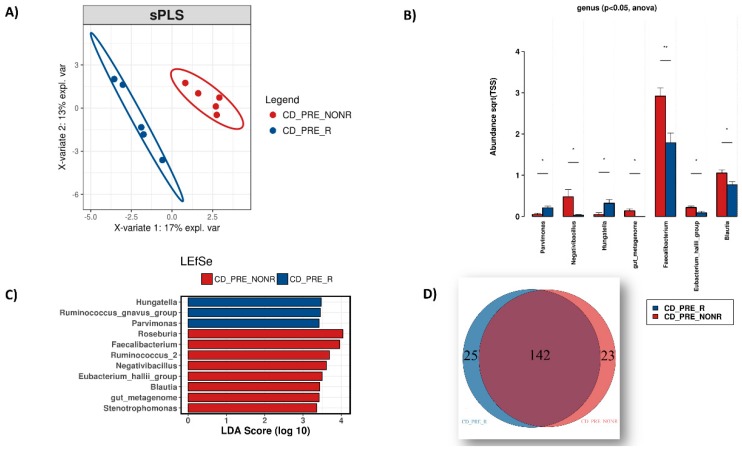
Microbiome analysis of Crohn’s disease samples before treatment of which we know the response outcome: response (CD_PRE_R) and non-response (CD_PRE_NONR). (**A**) β-diversity (qualitive enterotype differences). (**B**) Relative abundance changes of the microbial genera (**C**) LEfSe analysis showcasing microbial genera associated with the sample groups. (**D**) Venn diagram depicting the microbial genera constantly present (core microbiome) in the samples of the 2 groups.

We conducted several analyses on the 10 samples before treatment, dividing them into CD pre-treatment responders (CD_PRE_R) and CD pre-treatment non-responders (CD_PRE_NONR). Beta-diversity indicated distinct enterotypes for the two groups (Figure 6A) and differential abundance analysis using ANOVA revealed statistically significant (*p* < 0.05) differences in several genera between these groups (Figure 6B). *Parvimonas* and *Hungatella* were found to be more abundant in patients who would respond to IFX, whereas *Negativibacillus*, *Faecalibacterium*, *Eubacterium_hallii*_group and *Blautia* were more abundant in patients who would not respond. The LEfSe analysis (Figure 6C) was in accordance with these results, further highlighting the *Ruminococcus_gnavus*_group to be associated with patients who will not respond to treatment and *Roseburia*, *Ruminococcus_2* and *Stenotrophomonas* with CD patients who are likely to respond. Finally, by comparing the core microbiome of the two groups (Figure 6D), we identified 25 bacterial genera unique in patients who will respond to anti-TNF treatment and 23 in those who will remain unresponsive. The complete list of these genera can be found in Supplementary Dataset S4.

The low number of available UC biopsies in this study (two pre-treatment responders and two pre-treatment non-responders) allowed us to only carry out differential abundance analysis using ANOVA (Supplementary Figure S4). Regardless, we identified several statistically significant taxa according to ANOVA (*p* < 0.05). *Sphingomonas* had higher abundance in patients who would not respond to anti-TNF treatment, whereas *Sutterella*, *Ruminococcaceae_NK4A214_group*, *Roseburia*, *Proteus*, *Oribacterium*, *Merdibacter*, *Lactobacillus*, *Lachnospiraceae_NK4A136_group*, *Lachnospiraceae_ND3007_group*, *Intestinibacter*, *Haemophilus*, *Fournierella*, *Flavoninfractor*, *Eubacterium_coprostanoligenes_group* and *Clostridium_sensu_stricto_1* genera were more abundant in patients who will respond to treatment.

### 3.4. Differential Gene Expression Analysis (DGEA) of Mucosal Tissue

A focused immune/inflammation-related differential gene expression analysis was performed in the same set of biopsies and identified several differentially expressed genes in these samples. Comparison of gene expression levels between responder and non-responder CD at baseline identified 53 genes displaying reduced expression in biopsies from non-responders, including *TNFSF14, CCR7*, *CXCL8* and *NR3C1*, and seven genes with higher expression levels in non-responders, including *CCL22*, *CCL13* and *CCL11*.

Interestingly, following IFX treatment, non-responders displayed a higher number of upregulated genes compared to responders (58 versus 13). These included various TLRs and related molecules (*TLR1*, *TLR2*, *TLR3*, *TLR5*, *TLR6* and *MYD88*), chemokines and chemokine receptors (*CXCL1*, *CXCL3*, *CCR7*, *CCL4*, etc.), cytokines (*IL9*, *IL10*, *IL5*, *IL15* etc.), including TNF and COX-2 (*PTGS2*).

The complete list of pairwise analyses is shown in Supplementary Dataset S5.

### 3.5. Microbiome – Host Gene Expression Associations

To address putative microbiota–host gene expression associations, we performed correlation analysis of the microbiota and gene expression profiles of responders versus non-responders using Spearman’s Rho test. Thus, the genera that were differentially detected at baseline between responders versus non-responders, namely *Parvimonas*, *Hungatella*, *Negativibacillus*, *Faecalibacterium*, *Eubacterium_hallii*_group and *Blautia* were analysed vis-à-vis the differentially expressed inflammation-related genes. Supplementary Figure S5 presents a heatmap of this analysis for the responders at baseline and Supplementary Figure S6 for the non-responders.

Considering the differential gene expression at baseline between responders and non-responders (only genes with a Fold Change ≥15), we proceeded to examine the genera that are overabundant in responders. *Parvimonas* exhibits high positive correlation with *CXCL8*, *TLR6*, *TLR9*, *TNFSF14* and *SELE* and a strong negative correlation with *CCL22*, *IL18*, *IL15*, *CCR3*, *CD40LG*, *CSF1*, *NOS2*, *NR3C1*, *CCL8* and *CXCL2*. In non-responders, the only strong positive correlations identified for *Parvimonas* was with *CCR4* and *CXCL8*. *Hungatella* correlates positively with *CCR7*, *CXCR2*, *CXCL8*, *TLR6*, *TLR9*, *TNFSF14* and *SELE* and negatively with *CCL22*, *IL18*, *IL15* and *CCR*3. In non-responders, it correlates strongly with *CCL8*, *CCR3*, *CD40LG*, *CSF1*, *CXCL2*, *IL15*, *IL18*, *IL6R*, *NOS2*, *NR3C1*, *CCR7*, *TLR9*, *CCR4*, *CXCR2*, *TNFSF14*, *SELE* and *CXCL8* and negatively with *CCL22*. Thus, a pattern emerges where, out of 19 genes correlated with these genera, eight (*CXCL8, TLR6, TLR9, TNFSF14, SELE, IL18, IL15* and *CCR3*) are upregulated and one (*CCL22*) downregulated in responders. This microbial taxa and host gene expression combination can further allow us to distinguish responders from non-responders.

For the less abundant genera in responders, we observed that they also follow a similar pattern. *Negativibacillus* was positively correlated with *IL18, IL15, CCR3, CD40LG, CSF1, NOS2, NR3C1, CCL8, CXCL2,* and negatively with *CXCR2, CXCL8, TLR6, TLR9* and *TNFSF14* in responders, whereas it is negatively correlated only with *CCR4, CXCR2* and *CCR7*. With respect to *Faecalibacterium*, in responders there are strong positive correlations with *IL6R, CCL22, IL15, IL18* and *CCR3* but negative correlations with *CCR4, CCR7, CXCR2, CXCL8, TLR6, TLR9, TNFSF14* and *SELE*. In non-responders, there are only negative correlations with *TLR6* and *CXCL8*. For the *Eubacterium_hallii*_group, in responders there are only positive correlations with *CD40LG, CSF1, NOS2, NR3C1, CCL8, CXCL2, CCR9* and *CCR7*. For non-responders, we observed positive correlation with *CCR4* and *CXCR2* and negative for *CXCL8*. Finally, for *Blautia*, strong positive correlations in responders were observed for *IL18, IL15, CCR3, CD40LG, CSF1, NOS2, NR3C1, CCL8* and *CXCL2* and negative correlations for *CXCR2, CXCL8, TLR6, TLR9* and *TNFSF14*. For non-responders, there were only negative correlations with *CCR4, CXCR2* and *CCR7*.

Thus, out of 20 genes correlated with at least three out of four of these genera, we highlight nine (*IL18, CCR3, CXCL2, CXCR2, CXCL8, TLR6, TLR9, TNFSF14, CCR7*) upregulated in responders and two (*CCR4, CXCR2*) downregulated in non-responders. Interestingly, *CXCR2* appears to be downregulated in at least three out of four taxa in non-responders and upregulated in at least three out of four taxa in responders. These findings allow us to distinguish responders from non-responders.

**Figure 7:**
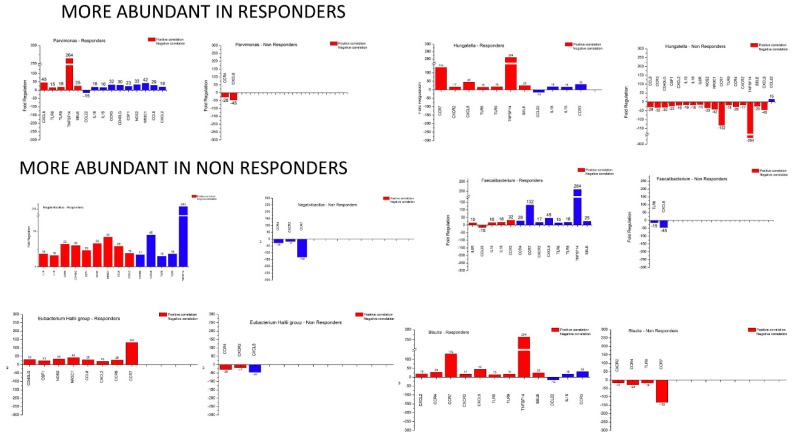
Overview of the genera with differential abundance between responders and non-responders and how these correlate with specific differentially expressed genes. IL18, CCR3, CXCL8, TLR6, TLR9 and TNFSF14 are upregulated and CCR4 is downregulated in both groups (2/2 genera abundant in responders and 3/4 abundant in non-responders) and appear to meet the criteria to be characterised as biomarkers for prediction of response to IFX therapy.

In summary (Figure 7), from the 60 DEGs between responders and non-responders and the six genera which appear to be differentially abundant in these groups, 19 DEGs were found to be correlated across all genera. Specifically for responders, six genes (*IL18, CCR3, CXCL8, TLR6, TLR9, TNFSF14*) are upregulated and one gene (*CCR4*) is downregulated in relation to the bacterial groups discussed earlier (two out of two taxa overabundant in responders and three out of four taxa overabundant in non-responders) and appear to meet the criteria to be characterised as biomarkers for prediction of response to IFX therapy.

## 4. Discussion

Inflammatory bowel diseases are attributed to genetic and non-genetic factors that intertwine to affect disease pathogenesis. In the present study, we have addressed host inflammatory gene expression and the mucosa-associated microbiota in the context of IFX response prediction.

Accumulating evidence underscores the impact of exaggerated inflammation and dysbiosis on the development of IBD [3]. The data presented in this study confirm previously reported changes in the biodiversity and composition of microbiota in IBD patients versus healthy controls [8,37,38]. Our analyses have focused on mucosal gut microbial communities as they are likely to be more relevant to IBD, for example, by interacting directly with and shaping the local immune cell repertoire, compared to luminal or fecal bacteria [39]. Indeed, bacterial diversity in the colon mucosa is under-represented in feces [40]. Unfortunately, this approach also prohibits us from detecting low-burden infections from bacteria that, for example, reside in mesenteric lymph nodes like the *Mycobacterium avium paratuberculosis,* which has been implicated in previous IBD studies [41,42], even though we detected an increase in other *Actinobacteria* like *Corynebacterium*.

We have found that α-diversity is reduced in the mucosa of IBD patients and that β-diversity is associated with reductions in the abundance of *Parabacteroides* [43,44], *Barnesiella* [45], *Butyricimonas* [46] and others. Furthermore, LEfSe analysis implicates some IBD-associated genera such as *Ruminococcus gnavus* as putative disease biomarkers [47].

It has been shown that various anti-cancer therapies, including chemo- and immuno-therapeutics, can cause changes in the gut microbiome which may in turn influence the outcome of treatment [48]. Patients receiving chemotherapy are also more vulnerable to opportunistic pathogens primarily due to compromised host immunity and deregulated intestinal microbiota [49]. Pre-clinical studies in mouse models have demonstrated that the gut microbiome influences the outcome of cancer immunotherapy [50,51]. Recent data expand these experimental findings to melanoma patients by identifying microbial signatures associated with response to anti-PD1 immunotherapy [52]. Our results demonstrate that anti-TNF therapy by IFX leads to dramatic shifts in certain bacterial genera in CD and to a reduction in α-diversity. Our observation aligns with a recently published study reporting changes in gut microbiota of IBD patients treated with Ustekinumab, a monoclonal antibody directed against the shared p40 subunit of IL-12 and IL-23 [53].

Furthermore, studies on rheumatology related disorders such as ankylosing spondylitis [54] and rheumatoid arthritis [55], which share a common inflammatory molecular background with IBD, have shown prior involvement of the microbiome in disease progression and treatment with anti-TNF. Involvement of bacterial genera like *Blautia*, which is highlighted in our study and its high abundance correlated with the non-responder’s group, was shown to be associated with positivity of rheumatoid factor (RF) or anti-citrullinated peptide antibodies [56]. In turn, the titer of anti-citrullinated peptide antibodies has been shown to have a predictive factor to anti-TNF therapy response [57].

The identification of eight bacterial genera that are detected only in IBD patients and not in HC and are either eliminated or reduced to non-detectable levels after IFX treatment, regardless of patient response, requires further studies to determine whether they represent opportunistic pathogens that colonize damaged and inflamed colonic mucosa and to characterise the mechanism by which TNF therapy leads to their elimination.

Given the known side-effects and associated costs of biological therapies, significant efforts have been channelled towards the discovery of biomarkers that will enable the prediction of response prior to initiation of treatment. Notwithstanding the complex nature of patient response to therapy [11,58,59], we reasoned that microbial signatures may serve as potential biomarkers of response to IFX. We have herein identified enterotypes that, at baseline, correlate with different responses to anti-TNF therapy. Thus, high abundance of *Blautia*, *Faecalibacterium*, *Roseburia* and *Negativibacillus* genera in CD patients before treatment is associated with disease refractory to IFX. Intriguingly, both *Faecalibacterium (prausnitzii)* and *Roseburia (intestinalis)* have been reported to confer anti-inflammatory properties in CD [60]. We hypothesize that their relative abundance in a subset of CD patients may be associated with pathogenic mechanisms which are not critically dependent on TNF. In contrast, a high abundance of *Hungatella, Ruminococcus gnavus* and *Parvimonas* at baseline typifies responsive patients. We propose that, together, these genera may serve as potential predictive biomarkers for therapeutic response to IFX. In UC, although the conclusions of our analyses become limited by the small sample size, the abundance of *Sutterella, Roseburia* and *Intenstinibacter* in responders indicates these taxa as candidates for further investigation.

We note that the enterotypes of both responding and non-responding patients change after IFX therapy. Our results highlight several taxa, such as *Anaerostipes, Eubacterium Halli, Escherichia Shigella* and *Butyricicoccus,* that are more prevalent in CD patients who have responded to treatment. In particular, the pathogenic *Shigella* is an interesting finding that points to an opportunistic infection. In UC, α-diversity appears to be reduced in responders and increased in non-responders after treatment, but interpretation is restricted by the small sample size. At phylum level, *Bacteroidetes* is reduced in UC relative to healthy controls but it is restored following IFX treatment in responders. This observation is in line with the contribution of endogenous TNF in TNBS-induced colitis in the mouse [61,62]. Our data also indicate that the UC responders may exhibit different enterotypes compared to non-responders and several taxa were uniquely associated with each group. The increase in *Ruminococcus torques* in responders is especially intriguing because previous work [63] has associated this genus with dysbiosis during IBD. There are clearly some limitations to studies of this nature regarding the conclusions the community can draw. Notably, since most of the CD samples analysed herein were taken from patients with non-structuring, non-penetrating CD (B1), fewer differences in microbiota composition versus healthy controls can be detected, as previously noted [8]. In addition, the small number of UC samples with the added effect of multiple group segmentation does not allow us to provide definitive results but rather indications towards IFX effects and response prediction. Finally, as is the case with most metagenomic studies, our work is also reliant on the rRNA databases and their annotations (note the gut_metagenome “genus” in some of the results), which we have addressed using the latest bioinformatics tools and databases.

Regarding host gene expression, several differentially expressed genes identified herein have previously been implicated in IBD pathogenesis [64,65,66,67,68]. Thus, *TNFSF14, CCR7* and *NR3C1* were found to be downregulated in the non-responders group at baseline. This finding aligns with the reported exaggerated colitis induced by dextran sodium sulfate in *Tnfsf14*^−/−^ compared to wild-type mice, indicating a protective role for TNFSF14 [67]. *Δ*ARE/CCR7^−/−^ mice also develop exacerbated ileitis and multiorgan inflammation [69]. Moreover, it has been reported that Foxp3^+^ Treg cells lacking *NR3C1* failed to prevent the induction of IBD in an in vivo mouse model [70]. *CXCL8* gene expression is closely correlated with the severity of inflammation, thus the different expression levels observed in CD responders versus non-responders may be due to differences in disease severity among patients, or due to the previous steroid treatment that results in decreased expression of CXCL8 [71]. Several chemokines were found to be upregulated in the non-responders group at baseline, which aligns with previous studies linking elevated chemokine levels to disease activity [72]. Anti-TNF treatment has a profound effect on mucosal gene expression in IBD patients [73]. We have found that following IFX treatment, non-responders displayed a higher number of upregulated genes compared to responders, including TLRs, chemokines and chemokine receptors, cytokines and other inflammatory mediators suggesting persistence of inflammation.

Recent studies have shown a crucial role for gut microbiota in regulating host gene expression [74,75]. Regarding IBD, Hasler et al. [76] reported that the expression levels of genes which are differentially expressed in IBD versus healthy subjects correlate with the abundance of microbial taxa but only during the healthy state and the correlation was missing in IBD patients. However, Magnusson et al. [77] reported that responders and refractory to anti-TNF therapy patients display distinctly separate patterns of mucosal antimicrobial peptide expression and gut microbiome before treatment. Thus, the interplay between anti-TNF therapy, host-gene expression and the microbiome in the intestinal mucosa remains poorly explored, albeit likely of importance for treatment outcome. In this study, we showed that the response to anti-TNF therapy is related to specific microbiota and inflammatory gene expression profiles in the gut. Correlation analysis between these parameters of response at baseline showed that the combined assessment of microbial taxa and host gene expression can further distinguish responders from non-responders.

Overall, our work provides the first evidence that certain enterotypes correlate with the response of CD patients to anti-TNF therapy and with patterns of host inflammatory gene expression. Combined, these findings could be further explored as prognostic indicators of a response to IFX therapy in larger patient cohorts from various geolocational and health-related backgrounds. Our results may also pave the way for modulation of microbiota composition via diet [78], probiotic and prebiotic products [79] or fecal microbiota transplantation [80,81] that could contribute towards improving the clinical benefits of anti-TNF therapy in IBD.

## Supplementary Materials

All supplementary materials of this work, for the purpose of this review only, can be found online at: https://drive.google.com/drive/folders/1i1gdsJ0Z1yA4_yVtHdrwvozSk4lPwcps Supplementary Table S1: Clinical characteristics of patients with Crohn’s disease (CD) and Ulcerative colitis (UC). Supplementary Dataset S1: A complete list of the genera depicted in the Venn diagram of FIGURE 3C. These are the genera that are constantly present (core microbiome) in the samples of the 3 groups versus those found exclusively in HC, CD and UC. Supplementary Dataset S2: A complete list of the genera depicted in the Venn diagram of FIGURE 5C. These are the genera that are constantly present (core microbiome) in the samples of the 3 groups versus those found only in responders, non-responders and at baseline (before initiation of treatment). Supplementary Dataset S3: A complete list of the genera depicted in the Venn diagram of SUPPLEMENTARY FIGURE 2C. These are the genera that are constantly present (core microbiome) in the samples of the 3 groups versus those found only in UC responders, non-responders and before treatment. Supplementary Dataset S4: A complete list of the genera depicted in the Venn diagram of FIGURE 6D. These are the genera that are constantly present (core microbiome) in the samples of the 2 groups versus those found exclusively in CD responders and non-responders to IFX treatment at baseline. Supplementary Dataset S5: Excel file with colour coded tabs: brown = differential gene expression before and after treatment, green = differential gene expression at baseline between patients who will and will not respond to treatment. All tabs contain genes with an absolute Fold Change > 2. Supplementary Figure S1: A) Microbiota composition changes at phylum level among Ulcerative Colitis patients before treatment (UC_PRE) and after treatment non-responders (UC_NONR) and responders (UC_R). B) α-diversity (quantification of biodiversity) differences of the 3 groups. C) β-diversity (qualitative enterotype differences) of the 3 groups. Supplementary Figure S2: A) Relative abundance changes of microbial genera among Ulcerative Colitis patients before treatment (UC_PRE) and after treatment non-responders (UC_NONR) and responders (UC_R). B) LEfSe analysis of the 3 groups showcasing microbial genera associated with the sample groups. C) Venn Diagram depicting the microbial genera constantly present (core microbiome) in the samples of the 3 groups versus those found only in responders, non-responders and before treatment. Supplementary Figure S3: Microbial genera that are only present in the pre-treatment Crohn’s Disease (CD_PRE) and Ulcerative Colitis (UC_PRE) samples and not the Healthy Controls (control). Additionally, they seem to have their populations heavily diminished after treatment regardless of response. Supplementary Figure S4: Relative abundance changes of the microbial genera of Ulcerative Colitis samples before treatment of which we know the response outcome: response (UC_PRE_R) and non-response (UC_PRE_NONR). Due to the small size of the sample pool the rest of the tests could not be reliably performed. Supplementary Figure S5: Heatmap of the Spearman’s correlation analysis for responders at baseline. Correlations towards the red spectrum represent strong associations between microbial genera and inflammation/immunity related genes that are differentially expressed versus non-responders. Likewise, correlations toward the blue spectrum represent strong inverse associations. Supplementary Figure S6: Heatmap of the Spearman’s correlation analysis for non-responders at baseline. Correlations towards the red spectrum represent strong associations between microbial genera and inflammation/immunity related genes that are differentially expressed versus responders. Likewise, correlations toward the blue spectrum represent strong inverse associations.
