# Supplementary material for: The Interplay between Mucosal Microbiota Composition and Host Gene-Expression is Linked with Infliximab Response in Inflammatory Bowel Diseases
Source: Microorganisms. 2020 Mar 20;8(3):438. doi: 10.3390/microorganisms8030438 (PMC7143962; doi:10.3390/microorganisms8030438)
Supplement: Supplementary file 1 [file microorganisms-08-00438-s001.zip › microorganisms-745774-si/supp figure 1.docx]

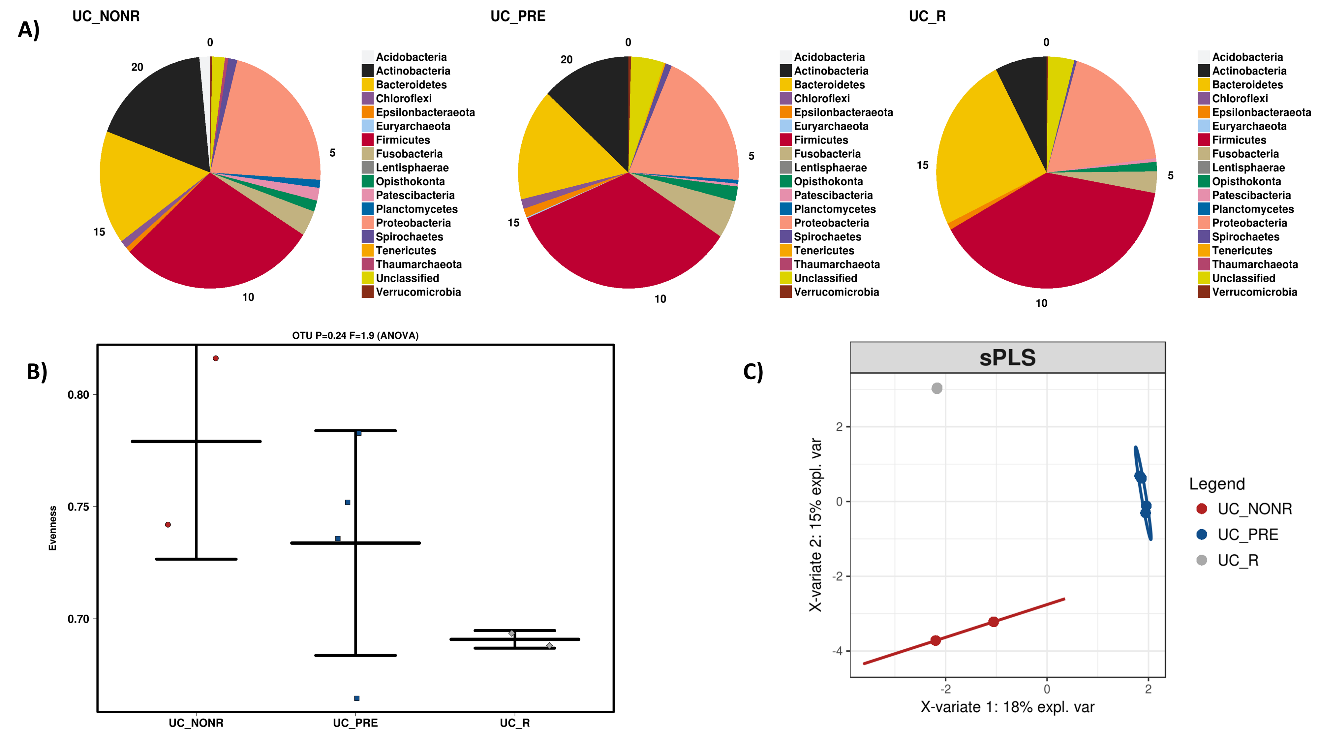


Supplementary Figure 1. A) Microbiota composition changes at phylum level among Ulcerative Colitis patients before treatment (UC_PRE) and after treatment non-responders (UC_NONR) and responders (UC_R). B) α-diversity (quantification of biodiversity) differences of the 3 groups. C) β-diversity (qualitative enterotype differences) of the 3 groups.
