# Supplementary material for: The Interplay between Mucosal Microbiota Composition and Host Gene-Expression is Linked with Infliximab Response in Inflammatory Bowel Diseases
Source: Microorganisms. 2020 Mar 20;8(3):438. doi: 10.3390/microorganisms8030438 (PMC7143962; doi:10.3390/microorganisms8030438)
Supplement: Supplementary file 1 [file microorganisms-08-00438-s001.zip › microorganisms-745774-si/Supp figure 3.docx]

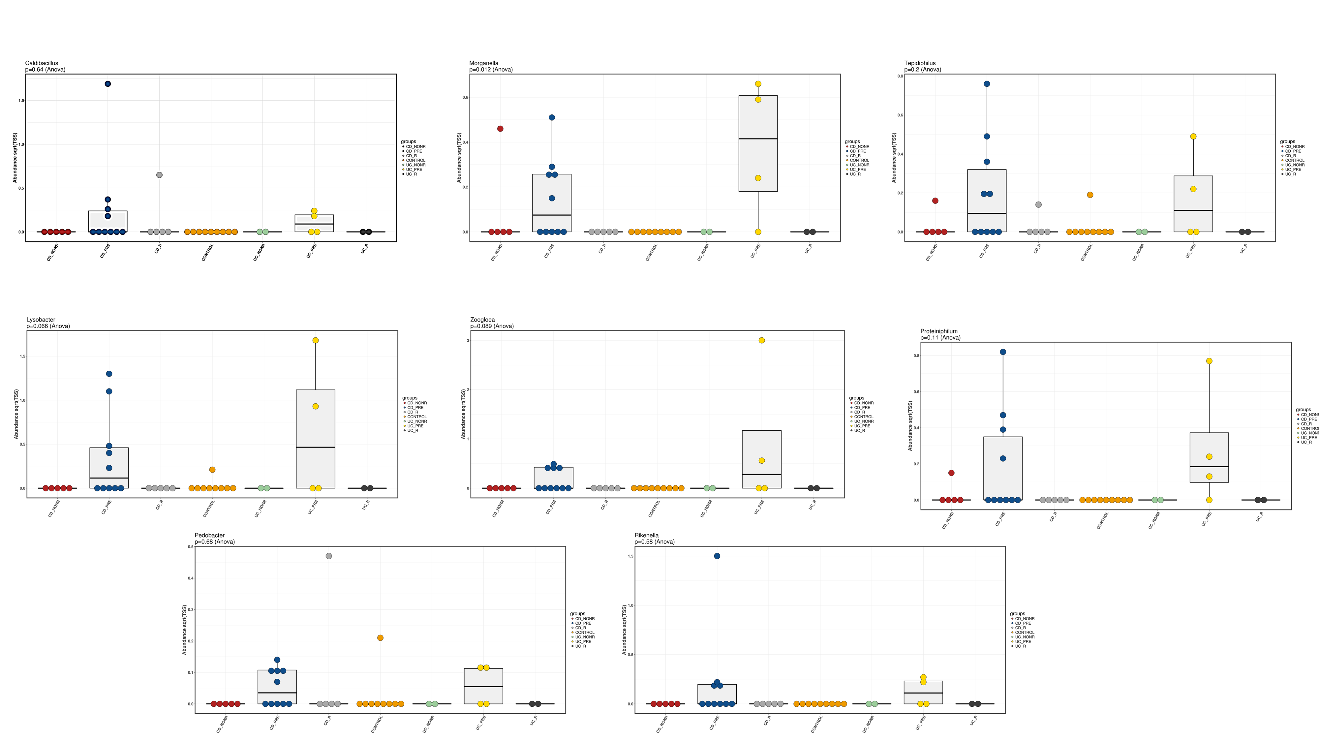


Supplementary Figure 3. Microbial genera that are only present in the pre-treatment Crohn’s Disease (CD_PRE) and Ulcerative Colitis (UC_PRE) samples and not the Healthy Controls (control). Additionally, they seem to have their populations heavily diminished after treatment regardless of response.
