# Supplementary material for: The Interplay between Mucosal Microbiota Composition and Host Gene-Expression is Linked with Infliximab Response in Inflammatory Bowel Diseases
Source: Microorganisms. 2020 Mar 20;8(3):438. doi: 10.3390/microorganisms8030438 (PMC7143962; doi:10.3390/microorganisms8030438)
Supplement: Supplementary file 1 [file microorganisms-08-00438-s001.zip › microorganisms-745774-si/supp figure 4.docx]

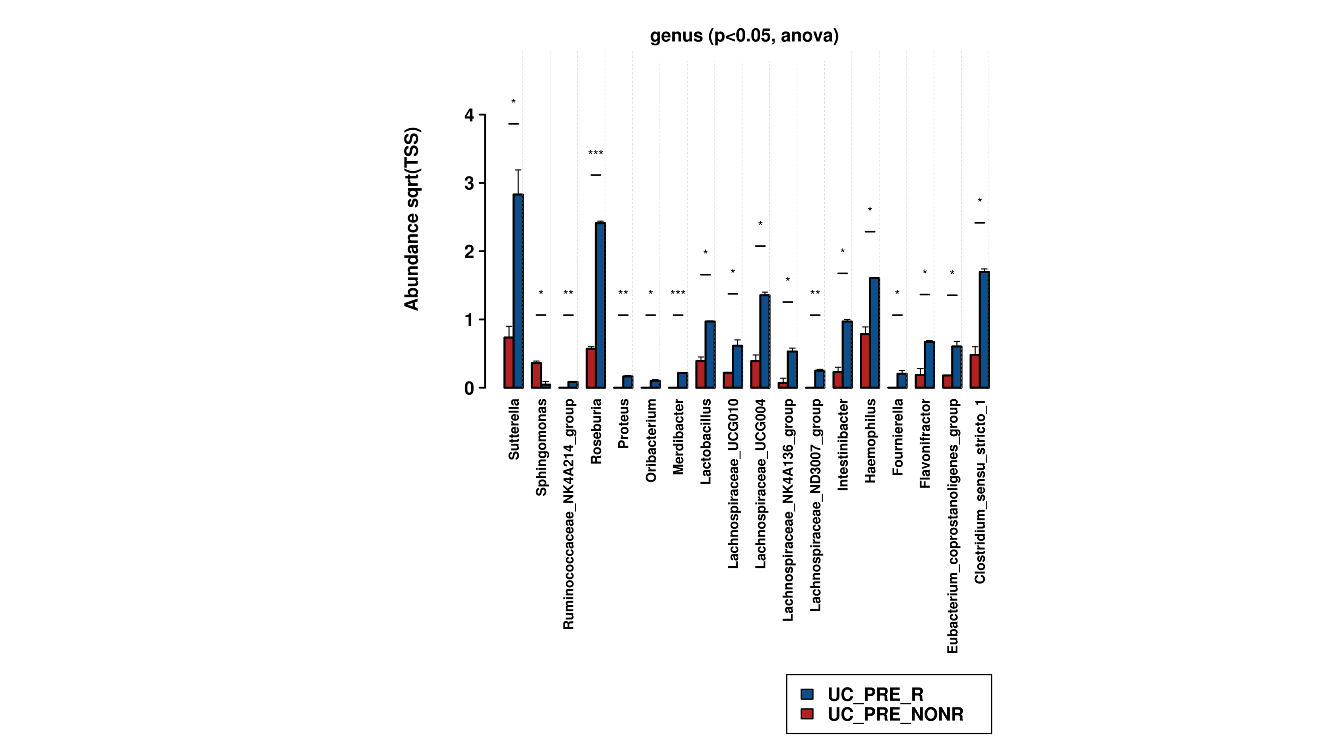


Supplementary Figure 4. Relative abundance changes of the microbial genera of Ulcerative Colitis samples before treatment of which we know the response outcome: response (UC_PRE_R) and non-response (UC_PRE_NONR). Due to the small size of the sample pool the rest of the tests could not be reliably performed.
