# Supplementary material for: The Interplay between Mucosal Microbiota Composition and Host Gene-Expression is Linked with Infliximab Response in Inflammatory Bowel Diseases
Source: Microorganisms. 2020 Mar 20;8(3):438. doi: 10.3390/microorganisms8030438 (PMC7143962; doi:10.3390/microorganisms8030438)
Supplement: Supplementary file 1 [file microorganisms-08-00438-s001.zip › microorganisms-745774-si/supp figure 5.docx]

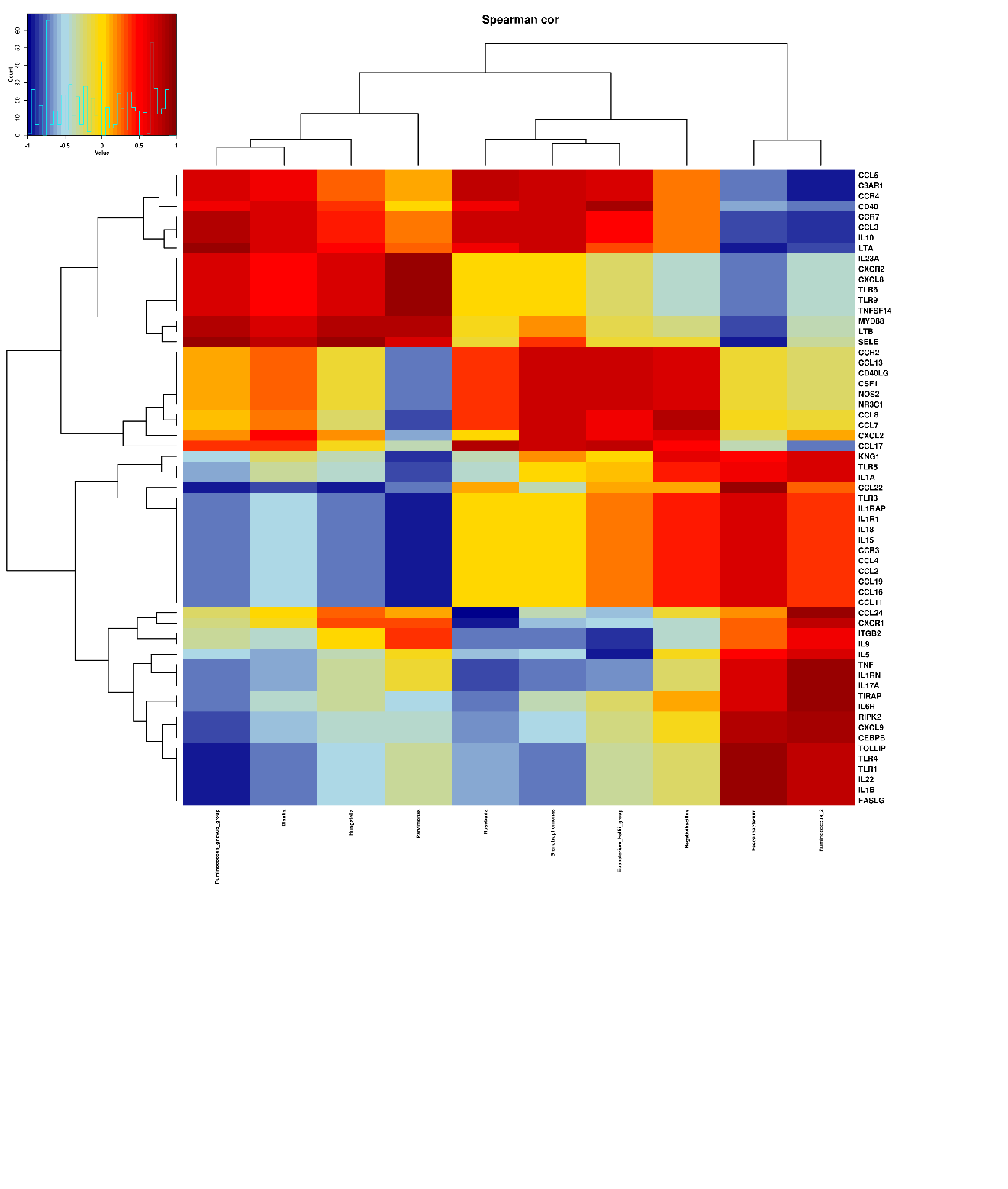


Supplementary Figure 5. Heatmap of the Spearman’s correlation analysis for responders at baseline. Correlations towards the red spectrum represent strong associations between microbial genera and inflammation/immunity related genes that are differentially expressed versus non-responders. Likewise, correlations toward the blue spectrum represent strong reverse associations.
