# Supplementary material for: The Interplay between Mucosal Microbiota Composition and Host Gene-Expression is Linked with Infliximab Response in Inflammatory Bowel Diseases
Source: Microorganisms. 2020 Mar 20;8(3):438. doi: 10.3390/microorganisms8030438 (PMC7143962; doi:10.3390/microorganisms8030438)
Supplement: Supplementary file 1 [file microorganisms-08-00438-s001.zip › microorganisms-745774-si/Supptable1.docx]

**Supplementary Table 1.** Clinical characteristics of patients with Crohn’s disease (CD) and Ulcerative colitis (UC)

| **Samples** | **Gender** | **Disease** | **Montreal Classification** | **Other Therapy before Ifliximab** | **HB index at baseline** | **CRP (mg/L) at baseline** | **Mayo total at baseline** | **HB index after treatment** | **CRP (mg/L) after treatment** | **Mayo total after treatment** | **Response to Infliximab** |
| --- | --- | --- | --- | --- | --- | --- | --- | --- | --- | --- | --- |
| 1 | Male | CD | A2L3B1 | No | 6 | 8.8 |  | 0 | 1.2 |  | Yes |
| 2 | Male | CD | A1L3B1 | Azathioprine | 6 | 7.2 |  | 0 | 2.36 |  | Yes |
| 3 | Male | CD | A3L3B1 | Azathioprine | 10 | 5.9 |  | 7 | 3.57 |  | No |
| 4 | Male | CD | A3L3B2 | Prednisolone | 10 | 22 |  | 6 | 3.1 |  | No |
| 5 | Male | CD | A2L3B1 | Prednisolone | 9 | 13.5 |  | 1 | 0.3 |  | Yes |
| 6 | Female | CD | A3L3B1 | No | 6 | 5.2 |  | 1 | 0.8 |  | Yes |
| 7 | Male | UC | E3 | Prednisolone |  | 1.2 | 3 |  | 0.3 | 0 | Yes |
| 8 | Male | CD | A3L3B1 | No | 7 | 2.3 |  | 0 | 0.2 |  | Yes |
| 9 | Male | UC | E3 | No |  | 5.3 | 3 |  | 5.2 | 8 | No |
| 10 | Male | UC | E2 | No |  | 39.5 | 2 |  | 31.2 | 12 | No |
| 11 | Female | CD | A3L3B1 | Azathioprine | 12 | 21 |  | 14 | 10.6 |  | No |
| 12 | Male | CD | A3L3B1 | 5-Azacytidine | 9 | 57 |  | 8 | 23 |  | No |
| 13 | Male | UC | E3 | No |  | 1.2 | 3 |  | 0.2 | 0 | Yes |
| 14 | Female | CD | A3L3B1 | No | 15 | 6.81 |  | 13 | 1.45 |  | No |
